# Medical Schools

**Published:** 1877-09-20

**Authors:** 


### CORRESPONDENCE.

"MEDICAL SCHOOLS.—*Messrs. Editors:*—Your occasional reference to the duty of medical men to aid in the work of elevating the standard of medical education, is understood and appreciated by the substantial men in the profession. But what can we do? Those of us who graduated twenty and thirty years ago in colleges where diplomas meant something, and carried with them some evidence of acquirement and efficiency in the various branches of medical science, are now surrounded by the hurried off-shoots of cheap medical schools, who know but little and care less for the dignity and honor of the profession. If we attack the schools at which they graduated, we incur their enmity and bitter prejudice, and make ourselves the targets for the envious shafts of numerous quacks and their friends. We say "quacks," for what else can they be called? Quacking is inculcated, we regret to say, in the very schools from which they emanate. It is the custom in one of these schools, which we could name, (a Southern school,) for the Professors to disregard the ethics in the matter of advertising themselves as specialists—send out their circulars to the people of successful and remarkable operations performed, while the school seeks notoriety through the village and backwoods newspapers, offering special inducements of cheapness, etc., to secure numbers, while the requirements for matriculation and graduation are so easy that anybody, however ignorant, may enter and obtain *sheep-skins*, and go out to prey upon the public, to divide, and, in a great degree, destroy the emoluments to the worthy members of the profession, and to degrade and lower the profession into contempt and dishonor, so that many good men retire from the practice in disgust.

We confess that we see little hope of correcting the evil. We notice in your advertising department that a certain Northern school has set a noble example in lengthening the term of the sessions, and elevating the standard of medical education. This is well. Let the true men of the profession encourage this move. Let our medical societies take proper ground upon this subject, and let professional men everywhere send their pupils to first-class schools, and ignore the cheap, shoddy concerns. Anatomy stands at the very basis of a proper medical education, and yet, either from lack of material or incompetency of teachers—perhaps both—it is woefully slurred over in certain of our Southern schools. We hope that your journal and others will continue efforts to arouse the profession to a proper sense of the importance of raising the standard of medical education. It was a great disappointment to progressive men when the late convention of medical teachers at Philadelphia failed to accomplish anything. It is the more important that the medical journals should not slacken their efforts on this subject."

The above is from an intelligent medical gentleman residing in one of the upper counties of Georgia. The subject is an important one, and we trust his views will be read and appreciated. As journalists we fully appreciate what he has said, and will do what we can to remedy the evils alluded to. He is doubtless correct as to the remedy. If Professors of medical colleges violate the ethics they are not less amenable to censure than the private members of the profession; and the true men of the profession throughout the country should, especially in their organized capacity —in their societies and associations—discountenance the schools which are taught by incompetent Professors, and who employ so many unprofessional ways and means to advertise themselves and build up their cheap institutions. The fault is in the preceptors not doing their duty. Let every preceptor see to it that his students are instructed to attend first-class colleges, where the best facilities and the highest order of talents are to be found.

